# Lymphocytic Choriomeningitis Virus Seroprevalence among Urban Pregnant Women and Newborns, Philadelphia, Pennsylvania, USA, 2021

**DOI:** 10.3201/eid3203.250910

**Published:** 2026-03

**Authors:** Dustin D. Flannery, Caitlin M. Cossaboom, Timothy D. Flietstra, Alvaro Zevallos Barboza, Heather H. Burris, Karen M. Puopolo, Aridth Gibbons, Deborah L. Cannon, Inna Krapiunaya, Leanna Sayyad, Katrin S. Sadigh, Kami Smith, Joel M. Montgomery, Trevor Shoemaker, John D. Klena, Scott M. Gordon

**Affiliations:** Author affiliations: Children’s Hospital of Philadelphia, Philadelphia, Pennsylvania, USA (D.D. Flannery, A. Zevallos Barboza, H.H, Burris, K.M. Puopolo, S.M. Gordon); University of Pennsylvania Perelman School of Medicine, Philadelphia (D.D. Flannery, H.H. Burris, K.M. Puopolo, S.M. Gordon); Centers for Disease Control and Prevention, Atlanta, Georgia, USA (C.M. Cossaboom, T.D. Flietsra, A. Gibbons, D.L. Cannon, I. Krapiunaya, L. Sayyad, K.S. Sadigh, K. Smith, J.M. Montgomery, T. Shoemaker, J.D. Klena)

**Keywords:** Lymphocytic choriomeningitis virus, viruses, congenital viral infection, zoonoses, serology, pregnancy, United States

## Abstract

Lymphocytic choriomeningitis virus (LCMV) is a globally distributed rodentborne pathogen that can cause severe congenital infections. We conducted a retrospective cross-sectional seroepidemiologic study using remnant serum samples from pregnant women and newborns at 2 hospitals in Philadelphia, Pennsylvania, USA. We tested samples for LCMV IgG and IgM in 3 phases: a high-risk group determined by neighborhood deprivation index scores, a random sample of all birthing women, and a group with prenatally diagnosed neurologic malformations. We found LCMV IgG seroprevalence was 2.4% among 700 high-risk and 2.7% among 300 randomly selected pregnant women. Seroprevalence varied by hospital site, maternal race or ethnicity, and neighborhood deprivation level. All seropositive maternal samples were IgM-negative. Thirty-seven pregnant women carrying fetuses with neurologic malformations were seronegative. Our findings highlight the risk for LCMV exposure in urban settings and emphasize the need for pregnant women to avoid contact with rodents to prevent this rare but serious congenital infection.

Lymphocytic choriomeningitis virus (LCMV) is a rodentborne pathogen that can cause severe congenital infections (*1*). LCMV is commonly carried by the house mouse (*Mus musculus*) (*2*). The virus is a member of the Arenaviridae family and is distributed globally in urban and rural settings; however, the burden of infection is not well defined. Infections have been reported throughout North and South America, Europe, Asia, and Australia (*3*). 

In adult humans, LCMV infection typically is associated with asymptomatic or mild illness, but severe disease can occur in immunocompromised patients (*4*). The virus has disproportionate health effects on fetuses and newborns when infection occurs during pregnancy and can lead to chorioretinitis, intracranial calcifications, a variety of neurologic malformations, intellectual disability, neurodevelopmental problems, and fetal death (*1*,*5*–*7*).

Although LCMV is a devastating congenital human pathogen, sparse epidemiologic data describe domestic LCMV seroprevalence in the United States (*8*). Previous serosurveys of adult urban dwellers in the United States in the 1980s and early 1990s found an LCMV seroprevalence of 2%–5%, suggesting that LCMV exposure might be common (*9*,*10*). Data collected during 1984–1989 showed an average of 9% of house mice trapped across Baltimore, Maryland, USA, had detectable LCMV antibodies (*2*). Infected animals were identified at 6 of 8 sites, and LCMV seroprevalence among mice varied from 3.9% to 13.4%. The sampled site with the highest seroprevalence was an inner-city residential area where positive mice were clustered within city blocks and households, and that clustering correlated with estimates of mouse density (*2*).

Scant data on LCMV seropositivity among urban pregnant women or general cohorts of infants with neurologic malformations are available for the United States (*8*). Although rapid genetic sequencing has identified more causes of congenital neurologic malformations, most (50%–60%) remain idiopathic (*11*,*12*). One prior Chicago, Illinois, USA–based serosurvey of a limited number of children with congenital chorioretinitis showed that LCMV exposure in that group was common, supporting the notion that congenital LCMV might be underdiagnosed (*13*). Therefore, to understand current perinatal LCMV seroprevalence and its implications, we aimed to estimate LCMV seroprevalence among a targeted high-risk sample of pregnant women, and a random sample of women who gave birth in urban Philadelphia, Pennsylvania, USA. We also aimed to assess LCMV antibody transfer to newborns of LCMV seropositive birth mothers and determine LCMV seroprevalence among newborns with neurologic malformations and their birth mothers.

## Materials and Methods

### Study Design and Serum Sample Collection

We conducted a retrospective cross-sectional seroepidemiologic study of available remnant serum samples from pregnant women and newborns at 2 birth hospitals, Pennsylvania Hospital and Hospital of the University of Pennsylvania, and a freestanding children’s hospital special delivery unit, all located in Philadelphia. As part of routine clinical care at the 2 birth hospitals, at admission for delivery, pregnant women have blood drawn for rapid plasma reagin testing to screen for syphilis, per public health guidelines (*14*). We obtained residual serum from that testing from the clinical laboratory at the time it was scheduled for disposal. We linked residual serum samples to demographic and clinical data abstracted from medical records and then deidentified data for analysis. All data abstraction occurred before serologic testing, and the analyst responsible for clinical data was blinded to serologic testing results. We also obtained serum samples from pregnant women referred to the Children’s Hospital of Philadelphia (CHOP) Special Delivery Unit for childbirth because of known fetal neurologic malformations. CHOP Birth Defects Biorepository routinely collects newborn serum samples. We deidentified all specimens used in this study, and specimens could not be linked to specific patients. The study was reviewed and deemed non–human subjects research as a public health surveillance project by the institutional review boards at the University of Pennsylvania and CHOP, with a waiver of informed consent. This activity was also reviewed by the Centers for Disease Control and Prevention (CDC) and was conducted consistent with applicable federal law and CDC policy (e.g., 45 C.F.R. part 46.102(a)).

We conducted the study in 3 phases. For phases 1 and 2 (Figure), we included serum samples from pregnant women who were admitted for childbirth during January 1–December 31, 2021, and who had an available remnant serum sample in the biobank registry. At 1 of the 2 birth hospitals, we included available remnant cord blood serum samples from newborns of seropositive women to assess placental antibody transfer. 

**Figure:**
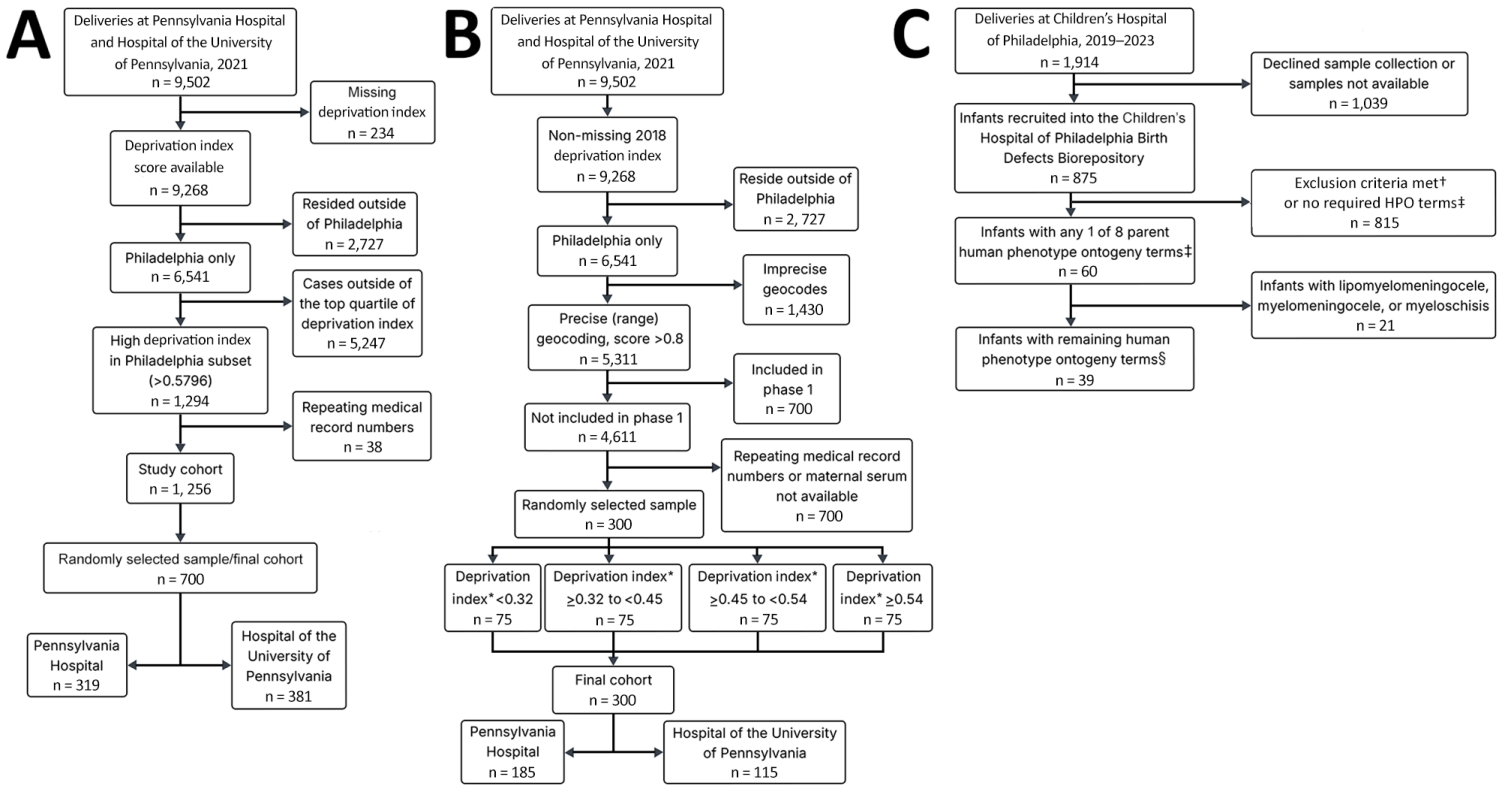
Flowchart of 3 phases of a study of lymphocytic choriomeningitis virus seroprevalence among urban pregnant women and newborns, Philadelphia, Pennsylvania, USA, 2021. A) Phase 1; B) phase 2; C) phase 3. Phases 1 and 2 used deliveries at Pennsylvania Hospital and the Hospital of the University of Pennsylvania in 2021 as the starting population. Repeated medical record numbers indicate parturient patients with multiple deliveries or >1 delivery during the study period, which would result in replicate maternal serum samples if retained. Phase 3’s starting population was the Children’s Hospital of Philadelphia Special Delivery Unit newborns delivered from the inception of the Birth Defects Biorepository during June 1, 2019–June 1, 2023. *Deprivation index ranges were determined using the Community Deprivation Index (https://github.com/geomarker-io/dep_index) on the basis of the geocoded address and quartile values were calculated on the full eligible cohort before phase-specific exclusions (n = 4,611). †Exclusion criteria included no cytomegalovirus testing on file, no placenta tissue available, and plasma not available for both maternal/infant matched samples even within the first few weeks after delivery. ‡The 8 overall HPO terms (https://hpo.jax.org) used encompassed 615 conditions. Those 8 HPO terms were abnormal choroid morphology, abnormal retinal morphology, cerebral calcification, intracranial cystic lesion, abnormality of neuronal migration, abnormal cerebral morphology, open neural tube defect, and abnormal cerebral ventricle morphology. §After excluding lipomyelomeningocele, myelomeningocele, and myeloschisis, 612 children’s HPO conditions remained. HPO, Human Phenotype Ontology.

For phase 1, we defined a high-risk group, assuming a likely association between residence in neighborhoods of lower socioeconomic status and higher mouse exposure (*15*). For that group, we selected serum samples from pregnant women residing in census tracts with the highest quartile of neighborhood deprivation according to the Community Deprivation Index (https://github.com/geomarker-io/dep_index) on the basis of the geocoded address reported at the time of admission for childbirth (*16*,*17*). Community Deprivation Index scores range from 0 to 1 and are divided into 4 quartiles from least deprived (Q1) to most deprived (Q4) as follows: Q1 is <0.324, Q2 is 0.324–0.449, Q3 is 0.45–0.579, and Q4 is >0.58 (*17*,*18*). The index was developed using a principal component analysis of the 2018 American Community Survey census tract fraction of the population with incomes below the federal poverty level, at least a high school education, lacking health insurance, receiving public assistance for income or food, median household income, and the fraction of vacant homes (*18*). 

For phase 2, to obtain a seroprevalence estimate for the study population at large, we selected serum samples from a random sample of pregnant women who gave birth during the study period. For phases 1 and 2, we excluded serum samples from pregnant women who had a missing deprivation index; resided outside of the Philadelphia city limits; had an imprecise geocode, defined as a geocode match score <0.8; or with <300 µL of available serum (Figure). We excluded samples from the 700 pregnant women in phase 1 from selection in phase 2 to avoid double selection considering that a large pool of samples remained and resampling was not necessary. We abstracted clinical data from the electronic medical record for all pregnant women with included serum samples.

For phase 3, we sought to estimate LCMV seroprevalence among a cohort of pregnant women with known fetal neurologic and ocular malformations and their newborns. We obtained maternal plasma and cord blood samples from the CHOP Birth Defects Biorepository for births during June 1, 2019–June 1, 2023. We used Human Phenotype Ontology (HPO) terms to define neurologic and ocular findings (*19*); those terms were developed to standardize the descriptions of phenotypes observed across human disease.

### LCMV Serologic and Molecular Testing

During September 2023–June 2024, we shipped samples from all 3 study phases to CDC (Atlanta, Georgia, USA) for laboratory testing. As previously described (*20*), the laboratory used a CDC-developed ELISA to determine the presence of LCMV IgG. The laboratory subsequently tested IgG-positive samples by using a CDC-developed IgM ELISA (*20*). For IgM-positive samples, the laboratory subsequently extracted RNA from the original clinical specimen and tested for LCMV nucleic acid using a real-time reverse transcription PCR targeting the large segment of LCMV (*21*).

### Statistical Analysis

For phase 1, from preliminary deprivation index data and historical childbirth volumes, we estimated that we would need 700 maternal serum samples to detect seroprevalence >0.5%: 500 samples for screening and 200 samples for validation to confirm the seroprevalence estimate. For phase 2, on the basis of preliminary findings from phase 1, we selected 300 random maternal samples stratified by deprivation index quartiles (75 samples per quartile) to obtain a seroprevalence estimate for the study population. For phase 3, we used a convenience sample, as described above. We reported the percentage of seropositive cases for each phase and computed 95% CIs by using the binomial distribution.

We used a 2-sample *z*-test for proportions and Fisher exact test to compare serostatus by available demographic data and clinical characteristics for phases 1 and 2 combined. Because phase 3 results were not comparable to phases 1 and 2, we did not include phase 3 in that analysis. To address potential collinearity, we used an analysis of variance (ANOVA) model with hospital, ethnicity, race, maternal age, and deprivation index quartile variables as the independent variables and serostatus as the response variable. Because we selected all samples from phase 1 from Q4, we used the deprivation index quartile in the ANOVA model to ensure that the overrepresentation did not bias the results. We then fit a multivariable logistic regression model with maternal LCMV serostatus as the binary outcome. We used stepwise variable selection to derive a final parsimonious model and calculated adjusted odds ratios (ORs) with 95% CIs. We used MATLAB (MathWorks, https://www.mathworks.com) to perform all statistical analyses and considered p<0.05 statistically significant.

## Results

In 2021, the 2 hospitals reported that 9,286 women gave birth to 9,502 newborns. For phases 1 and 2 combined, the median maternal age at delivery was 29 (IQR 24–34) years, and 109 (9.8%) patients gave birth before 37 weeks’ gestation. Of the 700 selected maternal serum samples from the high-risk group screened in phase 1, a total of 17 (2.4%, 95% CI 1.4%–3.9%) were positive for LCMV IgG, among which none were IgM-positive. We had 11 paired cord blood samples available from the 17 IgG-positive maternal serum samples, all of which tested positive for IgG directed to LCMV but were IgM-negative. 

In phase 2, a total of 8 (2.7%, 95% CI 1.2%–5.2%) of the 300 random maternal serum samples were IgG-positive for LCMV. None of those serum samples were IgM-positive. Six paired cord blood samples were available from the 8 IgG-positive maternal serum samples; all were IgG-positive for LCMV but were IgM-negative (Table 1).

**Table 1:** Phase-specific lymphocytic choriomeningitis virus seroprevalence among urban pregnant women, Philadelphia, Pennsylvania, USA*

Analysis phase	No. (%) seropositive
Pregnant women, n = 1,000	25 (2.5)
Phase 1, n = 700	17 (2.4)
Phase 2, n = 300	8 (2.7)
Pregnant women with antenatally diagnosed fetal brain anomaly; phase 3, n = 37	0

*Maternal lymphocytic choriomeningitis virus (LCMV) seroprevalence detected by LCMV IgG during pregnancy. For phases 1 and 2, neighborhood deprivation at the census tract level calculated by using the Community Deprivation Index (*17*,*18*). Scores are divided into 4 quartiles from least deprived (Q1) to most deprived (Q4). Scores are as follows: Q1 is <0.324; Q2 is 0.324–0.449; Q3 is 0.45–0.579; and Q4 is >0.58. Phase 1 included persons residing in the highest quartile of neighborhood deprivation; phase 2, included residents of all 4 quartiles of neighborhood deprivation. Phase 3 included persons with fetal brain anomalies diagnosed antenatally.

We compared serostatus by clinical and demographic characteristics for phases 1 and 2 combined (Table 2). The overall ANOVA model was statistically significant. The independent variables showed high collinearity in the initial model with an average variance inflation factor (VIF) of 1.57. The final ANOVA model (F statistic = 24.375; p = 0.0004) that used stepwise regression removed much of the collinearity from the initial model, resulting in an average VIF of 1.14. The statistically significant factors were the birth hospital (Pennsylvania Hospital ranked higher than Hospital of the University of Pennsylvania; p = 0.01), maternal race (all other races were higher than White race; p = 0.0003), and maternal ethnicity (Hispanic ethnicity ranked higher than non-Hispanic; p = 0.02). We additionally calculated ORs from those significant variables, as well as maternal age and deprivation index (Table 3). 

**Table 2:** Demographic and clinical characteristics of maternal patients in a study of lymphocytic choriomeningitis virus seroprevalence among urban pregnant women and newborns, Philadelphia, Pennsylvania, USA, 2021*

Characteristic	No. seropositive (%) [95% CI]	p value
Maternal age range, y		
14–23, n = 246	4 (1.6) [0.44–4.11]	0.2488
24–34, n = 570	19 (3.3) [2.02–5.16]	Referent
35–49, n = 184	2 (1.1) [0.13–3.87]	0.1268
Prepregnancy BMI†		
Underweight, BMI <18.5, n = 26	0 [0–10.88]	NA
Normal, BMI 18.5 to <25.0, n = 332	4 (1.2) [0.33–3.06]	0.3344
Overweight, BMI 25.0 to <30.0, n = 240	6 (2.5) [0.92–5.36]	Referent
Obese, BMI >30.0, n = 381	14 (3.7) [2.02–6.09]	0.4905
Group B S*treptococcus* status		
Positive, n = 295	4 (1.4) [0.37–3.43]	0.1695
Negative, n = 593	17 (2.9) [1.68–4.55]	Referent
Unknown, n = 112	4 (3.6) [0.98–8.89]	0.7600
Live-born infant		
Y, n = 995	25 (2.5) [1.63–3.69]	Referent
N, n = 5	0 [0–45.07]	NA
Gestational age at delivery		
<37 weeks, n = 108	3 (2.8) [0.58–7.90]	0.7836
>37 weeks, n = 892	22 (2.5) [1.55–3.71]	Referent
Birth hospital		
Pennsylvania Hospital, n = 504	17 (3.4) [1.98–5.35]	Referent
Hospital of the University of Pennsylvania, n = 496	8 (1.6) [0.70–3.15]	0.1036
Race‡		
Black, n = 655	22 (3.3) [2.12–5.04]	Referent
White, n = 243	1 (0.4) [0.01–2.27]	0.0086
Other, n = 86	1 (1.2) [0.03–6.31]	0.7154
Ethnicity§		
Hispanic, n = 153	7 (2.9) [1.17–5.84]	Referent
Non-Hispanic, n = 837	16 (1.9) [1.10–3.09]	0.0712
Neighborhood deprivation¶		
Q1, least deprived, n = 75	0 [0–3.92]	0.2406
Q2, n = 75	2 (2.7) [0.32–9.30]	1.0
Q3, n = 75	2 (2.7) [0.32–9.30]	1.0
Q4, most deprived, n = 75	21 (2.7) [1.68–4.11]	Referent

*BMI, body mass index; NA, not applicable. †Excludes 21 unknown, of which 1 was positive (not represented in table). ‡Excludes 16 unknown, of which 1 was positive (not represented in table). §Excludes 10 unknown, of which 2 were positive (not represented in table). ¶Neighborhood deprivation at the census tract level calculated by using the Community Deprivation Index (*17*,*18*). Scores are divided into 4 quartiles from least deprived (Q1) to most deprived (Q4). Scores are as follows: Q1 is <0.324; Q2 is 0.324–0.449; Q3 is 0.45–0.579; and Q4 is >0.58.

**Table 3:** Factors associated with maternal seroprevalence in a study of lymphocytic choriomeningitis virus seroprevalence among urban pregnant women and newborns, Philadelphia, Pennsylvania, USA, 2021*

Characteristic	Odds ratio (95% CI)
Maternal age range, y	
14–23	0.4793 (0.1614–1.4239)
24–34	Referent
35–49	0.3187 (0.0735–1.3814)
Birth hospital†	
Pennsylvania Hospital	2.1294 (0.9104–4.9802)
Hospital of the University of Pennsylvania	Referent
Race†	
Black	8.4107 (1.1275–62.7395)
White	Referent
Other	2.8471 (0.1761–46.0206)
Ethnicity†	
Hispanic	2.4602 (0.9948–6.0842)
Non-Hispanic	Referent
Neighborhood deprivation‡	
Q1, least deprived	NA§
Q2	0.9837 (0.2261–4.279)
Q3	0.9837 (0.2261–4.279)
Q4, most deprived	Referent

*NA, not applicable. †Statistically significant variable in final analysis of variance model. ‡Neighborhood deprivation at the census tract level calculated by using the Community Deprivation Index (*17*,*18*). Scores are divided into 4 quartiles from least deprived (Q1) to most deprived (Q4). Scores are as follows: Q1 is <0.324; Q2 is 0.324–0.449; Q3 is 0.45–0.579; and Q4 is >0.58. §NA indicates that no positive results were recorded within Q1, resulting in this odds ratio being undefined.

In phase 3, we assessed LCMV seroprevalence among a cohort of pregnant women carrying fetuses with prenatally diagnosed congenital malformations (Figure). We used HPO terms to identify neurologic or ocular malformations discovered prenatally or during an infant’s hospital stay. We tested for LCMV IgG in plasma from 37 pregnant women and cord blood samples from their 39 affected children (including 2 sets of twins) from June 1, 2019–June 1, 2023. Malformations included microcephaly (n = 17), ventriculomegaly (n = 10), corpus callosum abnormalities (n = 5), encephalocele (n = 2), retinal hemorrhage (n = 2), Dandy-Walker malformation (n = 1), absent septum pellucidum (n = 1), and posterior fossa cyst (n = 1). None of the samples from the 37 pregnant women or their 39 children were seropositive for LCMV IgG (Table 1; Figure); therefore, we did not test for IgM levels.

## Discussion

In this study of LCMV seroprevalence among a large and contemporary cohort of pregnant women in Philadelphia, we found that ≈2%–3% had evidence of exposure to the virus on the basis of detectable LCMV IgG. None of the seropositive samples had confirmed LCMV IgM, and cord blood samples from seropositive cases all had detectable LCMV IgG but no detectable LCMV IgM. Those findings suggest that ≈2–3 of 100 pregnant women in Philadelphia had past exposure to LCMV, although none had acute infection at the time of blood sample collection. The results also are consistent with passive antibody transfer from exposed pregnant women to their newborns because all cord blood samples from seropositive patients were also IgG positive. Our data provide insights into exposure of a large random sample of pregnant women and a targeted sample of high-risk pregnant women to a high-consequence and underrecognized congenital pathogen.

The seroprevalence estimates from this study align with data from other LCMV seroprevalence reports in the United States and worldwide (*8*). The largest prior LCMV serosurvey was conducted 35 years ago in urban Baltimore (*9*). That study included 1,149 persons who sought care at a sexually transmitted infections clinic. Among that patient cohort, 94% were African American (*9*), but pregnancy status was not reported. In that study, 54/1,149 (4.7%) patients were IgG seropositive (*9*), a modestly higher percentage than in our study. Those differences might be explained by differences in rodent exposure risk between the Baltimore and Philadelphia study populations or differences in LCMV carrier rates among mice in Baltimore versus Philadelphia during the study periods. Philadelphia is the sixth-largest US city and has >1.5 million inhabitants and ≈19,000 births annually (*22*). If our estimate of LCMV seroprevalence among pregnant women is generalizable to the overall population of Philadelphia, we can extrapolate that nearly 30,000–45,000 persons have been exposed to LCMV, including 400–600 pregnant women. Those data support that exposure to rodents shedding LCMV is common. Therefore, we support developing guidance for pregnant women to avoid rodent excreta to the extent possible, given risks of severe congenital disease, which can range from neurologic and ocular malformations to hydrops and fetal or neonatal death (*6*,*7*). Similar guidance is already in place to minimize risk of contracting other congenital pathogens, including *Listeria*, cytomegalovirus, *Toxoplasma*, *Treponema*, rubella, varicella, Zika, and others (*23*).

Although we hypothesized that residents in census tracts with higher neighborhood deprivation would have higher rates of LCMV seroprevalence compared with the random sample, that was not the case. We did not find a statistically significant difference between deprivation index quartiles, but the quartile with the least deprivation had zero seropositive samples identified among the 75 tested samples (Table 2). We acknowledge that census tract–level data might not accurately reflect a person’s risk for exposure to LCMV-infected rodents. Of note, the difference in seropositivity between the 2 hospitals might be explained by potential demographic and socioeconomic differences in the populations served. Larger cohorts, including patients and samples from multiple urban and rural areas, are needed to fully determine the relationship between specific housing and neighborhood characteristics and risk for LCMV exposure.

None of the cases of congenital neurologic malformations in phase 3 of this study appear to have been related to congenital LCMV infection, most likely because of the small sample size of infants with a variety of congenital neurologic malformations. None of those infants exhibited chorioretinitis, which is the common clinical feature among all reported live-born cases of congenital LCMV (*6*). Although the sensitivity of chorioretinitis in congenital LCMV remains to be determined in larger studies, we recommend that all infants with congenital neurologic malformations receive a thorough retinal exam and that those with evidence of chorioretinitis then be evaluated for LCMV antibodies. Larger cohorts of samples from affected newborns are needed to more accurately estimate the effects of LCMV infection during pregnancy on the fetus. In addition, prospective sampling of women before and during pregnancy will be required to associate seroconversion more definitively with adverse fetal outcomes. Efforts to increase awareness of LCMV infection during pregnancy and to educate clinicians about LCMV are warranted because of the potential for this virus to cause fetal neurologic disease.

Strengths of this study include the relatively large sample size of phases 1 and 2, identification of 3 populations of interest, and use of a detailed serologic testing strategy devised in collaboration with experts from CDC. The first limitation of this study is that using remnant samples could introduce selection bias; however, of the 9,502 deliveries from Pennsylvania Hospital and Hospital of the University of Pennsylvania in 2021, maternal samples were available for ≈8,739 (92%). Second, including samples from pregnant women residing in single urban area in a single year might mean results are not generalizable to other populations or later timeframes; however, LCMV seroprevalence would not be expected to change substantially within the same geographic area over short time intervals. Third, we used deprivation index data from 2018, and that temporal mismatch could lead to misclassification error. Fourth, we did not test all maternal samples for IgM in the absence of IgG, meaning that very early maternal infection might not have been detected. Finally, the CHOP Birth Defects Biorepository contained limited maternal and cord blood samples, leading us to test only for IgG in cord blood samples from infants born with congenital neurologic malformations and their respective maternal samples. In a prior case series, IgG titers were unequivocally positive at birth in infants with diagnosed congenital LCMV, and IgM might be absent in neonates. Although we did not definitively rule out early infection in this cohort, we do not suspect congenital LCMV in the absence of pathognomonic chorioretinitis and IgG seropositivity. Future prospective studies should be undertaken to clarify LCMV transmission dynamics and fetal risks among additional urban and rural populations to best inform clinical and preventive guidance.

In conclusion, our findings confirm the ongoing exposure of urban dwellers to LCMV and demonstrate the potential for maternal-to-fetal transfer of LCMV IgG. Our data update decades-old US prevalence estimates and highlight a continued risk of rodentborne virus exposure during pregnancy. Given LCMV’s potential to cause severe congenital disease, targeted public health messaging could help prevent exposure during pregnancy, especially in high-risk communities. We believe that LCMV risk should be included in standard education about congenital infections and that pregnant women and clinicians should be made aware of risks associated with LCMV infection during pregnancy.
